# Disruption of the oral microbiota is associated with a higher risk of relapse after allogeneic hematopoietic stem cell transplantation

**DOI:** 10.1038/s41598-021-96939-8

**Published:** 2021-09-02

**Authors:** Vinícius Campos de Molla, Vitor Heidrich, Julia Stephanie Bruno, Franciele Hinterholz Knebel, Wanessa Miranda-Silva, Paula Fontes Asprino, Luciana Tucunduva, Vanderson Rocha, Yana Novis, Anamaria Aranha Camargo, Eduardo Rodrigues Fregnani, Celso Arrais-Rodrigues

**Affiliations:** 1 Centro de Oncologia, Hospital Sírio Libanês, Rua Dona Adma Jafet, 91, São Paulo, SP 01308-050 Brazil; 2 Universidade Federal de São Paulo, São Paulo, SP Brazil; 3 Centro de Oncologia Molecular, Hospital Sírio Libanês, São Paulo, SP Brazil; 4 Departamento de Bioquímica, Instituto de Química, Universidade de São Paulo, São Paulo, SP Brazil; 5 Hospital das Clínicas da Faculdade de Medicina, Universidade de São Paulo/ICESP, Sao Paulo, Brazil; 6 Churchill Hospital, NHS-BT, Oxford, UK

**Keywords:** Translational research, Haematopoietic stem cells

## Abstract

Intestinal microbiota (IM) diversity and composition regulates host immunity and affects outcomes after allogeneic stem cell transplantation (allo-HSCT). We evaluated if the oral mucosa microbiota (OM) could impact the outcomes in patients who underwent allo-HSCT. Samples from the oral mucosa of 30 patients were collected at three time points: before the conditioning regimen, at aplasia, and at engraftment. We analyzed the associations of OM diversity and composition with allo-HSCT outcomes. Lower OM diversity at preconditioning was associated with a higher risk of relapse at 3 years (68% versus 33%, respectively; *P* = 0.04). Dominance (relative abundance ≥ 30%) by a single genus at preconditioning was also associated with a higher risk of relapse (63% versus 36% at 3 years, respectively; *P* = 0.04), as well as worse progression-free survival (PFS; 19% versus 55%, respectively; *P* = 0.01), and overall survival (OS) at 3 years (38% versus 81%, respectively; *P* = 0.02). In our study we observed that OM dysbiosis is associated with a higher risk of relapse and worse survival after allo-HSCT.

## Introduction

Allogeneic hematopoietic stem cell transplantation (allo-HSCT) remains the only therapeutic option for several hematological neoplasms^1^. Although transplant outcomes have markedly improved in recent decades, relapse of the underlying condition remains the leading cause of death after allo-HSCT^2^. Despite conflicting results, several risk factors have been shown to affect relapse, including the intensity of the conditioning regimen^3–5^, pre-HSCT disease status^6^, donor age^7,8^, graft source^9^, killer immunoglobulin-like receptor compatibility^10^, graft versus host disease (GVHD) prophylaxis^11–13^, and the occurrence of chronic GVHD (cGVHD)^14^. Infections, acute GVHD (aGVHD), cGVHD, and secondary neoplasia are the main causes of non-relapse mortality (NRM)^2,15^. The disease risk index (DRI) stratifies the risk of mortality in patients after allo-HSCT, according to diagnosis and disease status^16^.

The intestinal microbiota (IM) has been shown to play a vital role in regulating host immunity^17^ and improving antineoplastic activity^18,19^. In addition, IM disruption, characterized by significant changes in microbiota diversity and composition, is associated with allo-HSCT clinical outcomes. Common complications after allo-HSCT, such as infections, mucositis, and GVHD, are associated with significant changes in IM diversity and composition. In allo-HSCT, IM disruption is also associated with the incidence of GVHD^20–22^, overall survival (OS)^23–26^, and underlying disease relapse^27,28^.

The human oral cavity harbors the second most abundant microbiota after the gastrointestinal tract. As observed for the IM, the oral microbiota (OM) directly influences human health^29^. OM disruption has been observed in several diseases, including diabetes, autoimmune diseases, endocarditis, gastrointestinal cancer, head and neck cancer^30–32^, and acute lymphoblastic leukemia^33^. Changes in the OM in patients undergoing allo-HSCT are known to be associated with respiratory signs and symptoms^34^ and oral mucositis^35^; however, no correlation between OM and allo-HSCT outcomes have been reported to date.

Accordingly, in this study, we evaluated whether the OM disruption is related to outcomes in patients who underwent allo-HSCT.

## Methods

### Patient characteristics and sample collection

We collected samples from the oral mucosa of patients who underwent allo-HSCT at Hospital Sírio Libanês, São Paulo, Brazil between January 2016 and April 2018.

Samples were collected by rubbing the dorsal tongue and andwith sterile swabs at three time points: before the conditioning regimen and before the oral medicine specialist intervention (preconditioning), at aplasia (defined as the first day of neutrophils under 0.5 × 10^3^/uL), and at engraftment. All patients were requested not to perform oral hygiene for at least 6 h before collection. Informed consent was obtained from all patients before collection. The study was approved by the local ethics committee (Comite de Ética em Pesquisa—Hospital Sírio Libanês), according to the Declaration of Helsinki. No tissue was procured from prisoners in this study. All patients were examined by an oral medicine specialist for potential infections, and all followed the same protocol for oral mucositis prophylaxis with photobiomodulation and oral hygiene with fluoride toothpaste and 0.12% chlorhexidine mouthwash. The standard antimicrobial prophylaxis in our institution included oral levofloxacin, acyclovir, and antifungal prophylaxis according to the patient's risk of fungal infection (voriconazole for high-risk patients, and fluconazole for low risk patients).

### DNA extraction

Bacterial cells were recovered from oral mucosa swabs through vortexing in TE buffer supplemented with 6 μL PureLink RNAse A (20 mg/mL; Thermo Fisher Scientific, Waltham, MA, USA). DNA was extracted using a QIAamp DNA Blood Mini Kit (Qiagen, Hilden, Germany) according to the manufacturer’s protocol (DNA Purification from Blood or Body Fluids) and stored at − 80 °C.

### 16S rRNA amplicon sequencing

For 16S rRNA amplicon sequencing, 12.5 ng DNA and prevalidated primers^36^ were used to amplify 16S rRNA hypervariable regions V3–V4. Amplicons were sequenced as described elsewhere^37^ on an Illumina MiSeq platform (Illumina, San Diego, CA, USA).

### Bioinformatics pipeline

Reads were demultiplexed, and primer sequences were removed using the MiSeq Reporter software. Within the QIIME 2 framework^38^, using experiment-specific adaptive error models^39^, forward and reverse sequences were filtered for quality and bimeras, denoised, and merged into consensus sequences with the DADA2 pipeline^40^, generating unique amplicon sequencing variants (ASVs). ASVs were further filtered for chimeric sequences using the SILVA database^41^ and UCHIME^42^. ASVs were taxonomically assigned using SILVA database and VSEARCH tool^43^.

### Statistical analyses

For alpha diversity analyses, the samples were rarefied to 12,500 reads before calculating the Shannon index, Simpson index, or the number of observed ASVs as bacterial diversity measures with the QIIME 2 *q2-diversity* plugin. Alpha diversity across groups was compared with the Mann–Whitney U test. OM diversity was classified based on the median Shannon index diversity measure across the study population at a given collection time point. Patients were classified as high diversity (above the Shannon index median) and low diversity (below Shannon index median). Fisher’s exact tests and two-sided Student's t-tests were used to evaluate the associations between alpha diversity status and categorical and numerical clinical parameters, respectively. The relative abundance of each taxa was calculated with the QIIME 2 q2-taxa plugin. The taxa shown on relative abundance longitudinal plots are all those showing dominance (relative abundance ≥ 30%) in at least one study sample or relative abundance ≥ 5% in at least 25% of study samples. Differentially abundant genera across transplantation phases were identified using ANCOM test, with relative differences represented by the log-transformed average relative abundance fold change between groups. ANCOM W represents the proportion of null hypotheses rejected when sub-testing the differential abundance of a genus normalized by the abundance of each one of the genera in the dataset. W > 0.7 was considered as statistically significant. The relative abundance of a genus was considered to increase during allo-HSCT for a given patient when the relative abundance at engraftment was greater than at preconditioning and the final relative abundance was ≥ 0.1%. The probabilities of progression-free survival (PFS) and OS were calculated using the Kaplan–Meier method and compared using log-rank tests. Cumulative incidence rates were calculated for aGVHD, cGVHD, NRM, and relapse/progression. Ninety-five percent confidence intervals (95% CIs) were estimated using the Greenwood formula. Adjusted probabilities for outcomes after transplantation were estimated using the Cox proportional hazards method (PFS and OS) and Fine-Gray risk regression model (aGVHD, cGVHD, NRM, and relapse/progression). The association between OM parameters and HSCT outcome was investigated in the final model after adjusting for the DRI. First-order interactions between OM parameters and each variable of interest were examined. The results are presented as relative risks of failure (adverse prognostic factors versus good prognostic factors), with 95% CIs and two-tailed *P* values. To examine the association between genus presence at preconditioning and relapse, only genera present in 25–75% of samples were evaluated, where presence was defined as relative abundance ≥ 0.1%. R software (version 3.5.0) and RStudio (version 1.2.5033) were used for statistical analyses. The statistical package *cmprsk* was used to evaluate relapse across groups with transplant-related death as the competing risk.

### Ethics approval and consent to participate

The study was approved by the local ethics committee, according to the Declaration of Helsinki.

## Results

### Patient characteristics

Between January 2016 and April 2018, 30 patients who underwent allo-HSCT for hematologic malignancies and had oral mucosa samples collected were included in this study. The most common underlying diseases were acute myeloid leukemia and acute lymphoblastic leukemia (60%). Conditioning regimens and intensity, graft source, T-cell depletion, and other clinical characteristics are listed in Table 1. The underlying disease, disease status, and OM diversity at preconditioning are presented in Table S1. The median follow-up time for survivors was 41 (30–50) months.

**Table 1:** Clinical characteristics of the study patients.

	N = 30
Sex (male)	16 (53%)
Age in years (median, range)	50 (19–73)
**Underlying disease**	
Acute myeloid leukemia	18 (60%)
Acute lymphoblastic leukemia	7 (23%)
Non-hodgkin lymphoma	5 (17%)
Myelodysplastic syndrome	4 (13%)
Chronic lymphocytic leukemia	1 (3%)
Chronic myeloid leukemia	1 (3%)
Multiple Myeloma	1 (3%)
**Conditioning intensity**	
Reduced intensity	18 (60%)
Myeloablative	12 (40%)
Total body irradiation	11 (37%)
Pretransplant T-cell depletion	15 (50%)
**Graft source**	
Bone marrow	10 (33%)
Peripheral blood	20 (67%)
**Donor**	
Matched sibling	9 (30%)
Haploidentical	10 (33%)
Matched unrelated	9 (30%)
Mismatched unrelated	2 (7%)
**Pretransplant comorbidity (HCT-CI)**	
0	16 (53%)
1–2	8 (27%)
≥ 3	6 (20%)
**Disease risk index**	
Low–intermediate	17 (57%)
High	13 (43%)
**Disease status at transplant**	
First or second complete remission	22 (73%)
Third complete remission	2 (7%)
Partial remission or refractory disease	6 (20%)
**GVHD prophylaxis**	
MMF + CsA	11 (37%)
MTX + CsA	10 (33%)
MMF + CsA + PTCy	9 (30%)
Follow-up in months (median, range)	37 (25–46)

*MMF* mycophenolate mofetil, *MTX* methotrexate, *CsA* cyclosporin A, *PTCy* post-transplant cyclophosphamide.

### Microbiota dynamics analyses

In total, 5,920,836 high-quality bacterial assigned sequencing reads were analyzed, representing 1723 unique ASVs. Out of the 90 samples sequenced, nine were excluded from diversity analyses owing to an insufficient number of high-quality reads (< 12,500 reads per sample, as determined using alpha diversity rarefaction curves) after the read-filtering steps employed in the pipeline. Therefore, adequate preconditioning samples were available for 27 of the 30 patients included in this study.

**Figure 1:**
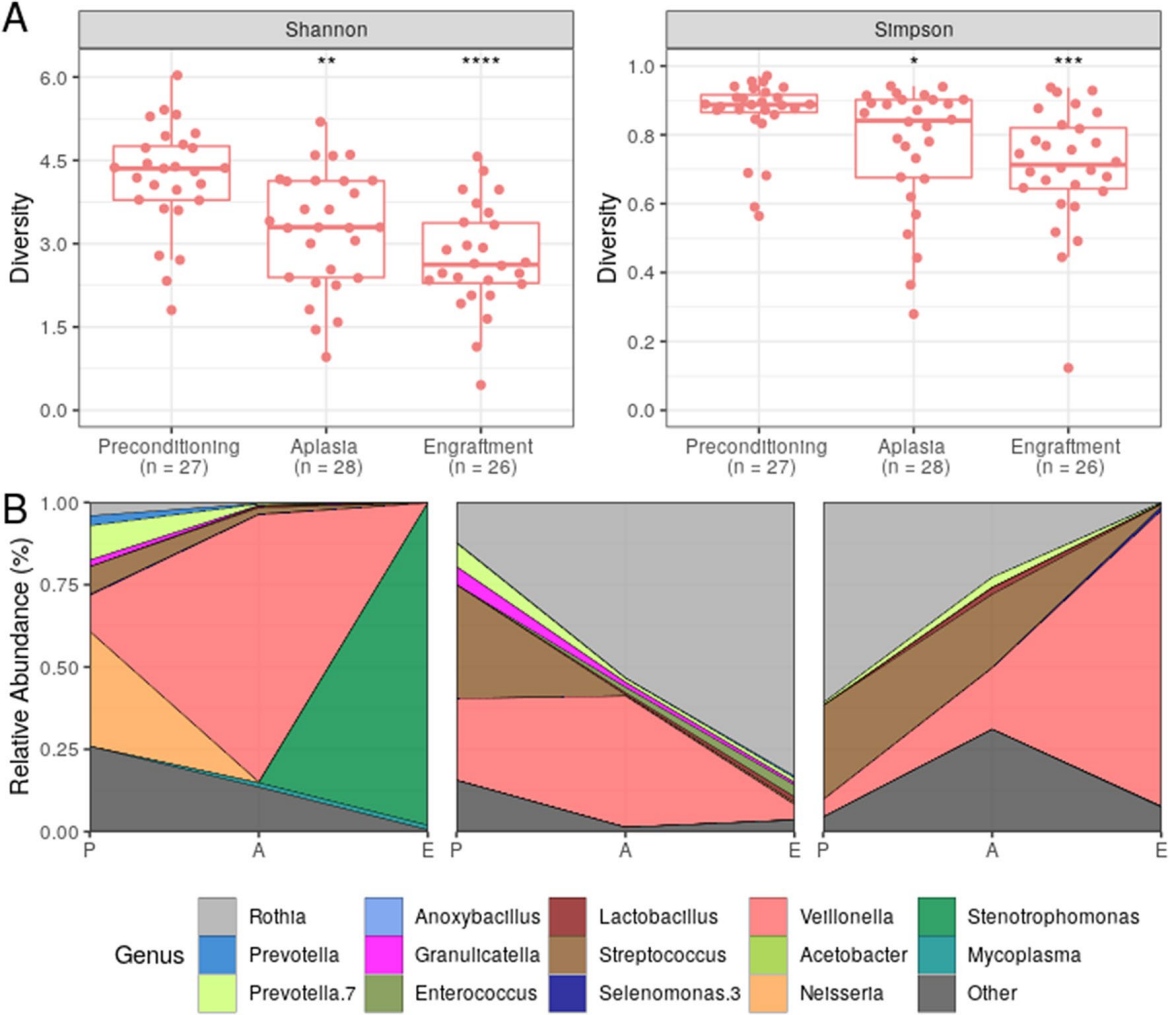
Bacterial diversity within the oral mucosa decreases during allo-HSCT. (**A**) Oral microbiota (OM) bacterial diversity boxplot at preconditioning (n = 27), aplasia (n = 28), and engraftment (n = 26) as measured by either Shannon index (left panel) or Simpson index (right panel). Mann–Whitney U tests were used with the preconditioning collection as the reference for comparisons. The boxes highlight the median values and cover the 25th and 75th percentiles, with whiskers extending to the more extreme value within 1.5 times the length of the box. Outliers are represented explicitly. Asterisks represent statistical significance: *P < 0.05; **P < 0.01; ***P < 0.001; ****P < 0.0001. (**B**) OM genera relative abundance composition across transplantation phases for three representative patients showing the decrease in bacterial diversity. Only genera showing relative abundance ≥ 30% in at least one study sample or relative abundance ≥ 5% in at least 25% of study samples are shown. *P* preconditioning, *A* aplasia, *E* engraftment.

The intrasample bacterial diversity (Fig. 1A) and richness (Fig. S1) of OM samples decreased significantly during the clinical course. This drop in diversity is associated with changes in taxa relative abundance during the same period (Fig. S2). Notably, all patients showed bacterial dominance by a single genus after preconditioning. In Fig. 1B, we show three representative patients with major dominance (relative abundance > 80%) by a single genus (*Stenotrophomonas*, *Rothia*, and *Veillonella,* respectively) at engraftment.

For a broader assessment of the relative abundance changes from preconditioning to subsequent transplantation phases, we employed the ANCOM test at the genus level. We observed statistically significant variations in the abundance of both opportunistic pathogenic and commensal genera (Fig. S3). From preconditioning to aplasia, there was a significant increase in the abundance of the potentially pathogenic genera *Enterococcus* and *Lactobacillus,* which were even more increased in the engraftment phase in terms of relative abundance fold change from preconditioning. *Staphylococcus* and *Mycoplasma* were other potentially pathogenic genera increased at engraftment. Contrarily, there was a significant decrease in the abundance of the commensal genera *Haemophilus* (at aplasia) and *Gemella* (at engraftment).

A global increase of potentially pathogenic genera occurs during allo-HSCT. However, evaluating each patient individually, we noticed irregular changes in the relative abundance of those same genera from preconditioning to engraftment. An increase in the relative abundance of *Enterococcus*, *Lactobacillus*, *Staphylococcus,* and Mycoplasma was observed in 32%, 40%, 56%, and 68% of patients (Fig. S4). Patients who presented an increase in *Enterococcus* relative abundance had a higher incidence of cGVHD when compared with patients without the increase of relative abundance (*P* = 0.03). No other associations between the increase in the relative abundance of potentially pathogenic genera and allo-HSCT outcomes was observed (Table S2).

### Impact of OM diversity on transplant outcomes

**Figure 2:**
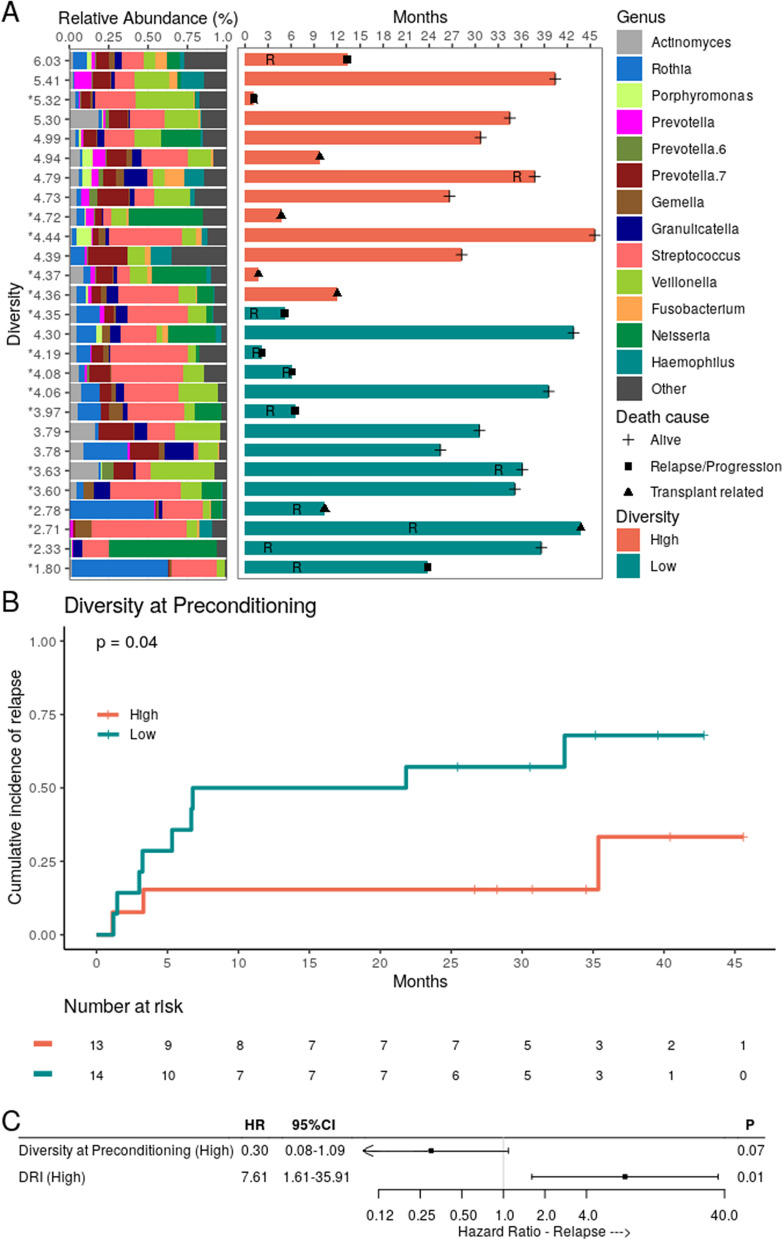
Oral microbiota bacterial dominance and bacterial diversity at preconditioning increased the risk of relapse in patients who underwent allo-HSCT. (**A**) Oral microbiota (OM) composition and diversity at preconditioning and the respective transplant course in each patient (n = 27). Patients are sorted based on descending Shannon diversity index, with the measures shown in the left subplot y-axis. The asterisk in the Shannon index indicates patients with at least one dominant (relative abundance > 30%) genus at preconditioning. Only genera showing relative abundance ≥ 30% in at least one preconditioning sample or relative abundance ≥ 5% in at least 10% of preconditioning samples are shown. Relevant outcomes (relapse and death) after infusion (aplasia) are shown in a timeline (in months) subplot (right). The plus sign represents censoring. *R* relapse. (**B**) Cumulative incidence of relapse with patients (n = 27) stratified by OM bacterial diversity at preconditioning (high versus low). (**C**) The DRI-adjusted hazard ratio for the association of OM bacterial diversity at preconditioning and relapse (n = 27).

In order to elucidate the impact of OM bacterial diversity on allo-HSCT outcomes, we stratified patients into low or high diversity at each collection time (Table S3). A swimmer plot was used to illustrate these correlations at preconditioning (Fig. 2A). When we compared those with high or low OM diversity at preconditioning, no differences were found in PFS (36% versus 32%, respectively; hazard ratio [HR] 0.75, 95% CI 0.28–2.00, *P* = 0.57), or in OS at 3 years (54% versus 57%, respectively; HR 0.96, 95% CI 0.33–2.89, *P* = 0.96). We also did not observe any differences in aGVHD at 100 days (43% versus 62%, respectively; HR 1.77, 95% CI 0.66–4.81, *P* = 0.26). At 3 years, no difference between high and low diversity in the incidence of cGVHD (30% versus 7%, respectively; HR 4.79, 95% CI 0.56–40.8, *P* = 0.15), and NRM (18% vs. 0%, respectively, HR 4.12, 95% CI 0.86–19.32, *P* = 0.07). However, lower OM diversity at preconditioning was associated with a higher risk of relapse at 3 years when compared with higher diversity (68% versus 33%, respectively; HR, 95% CI, P = 0.04; Fig. 2B, Table S4).

**Figure 3:**
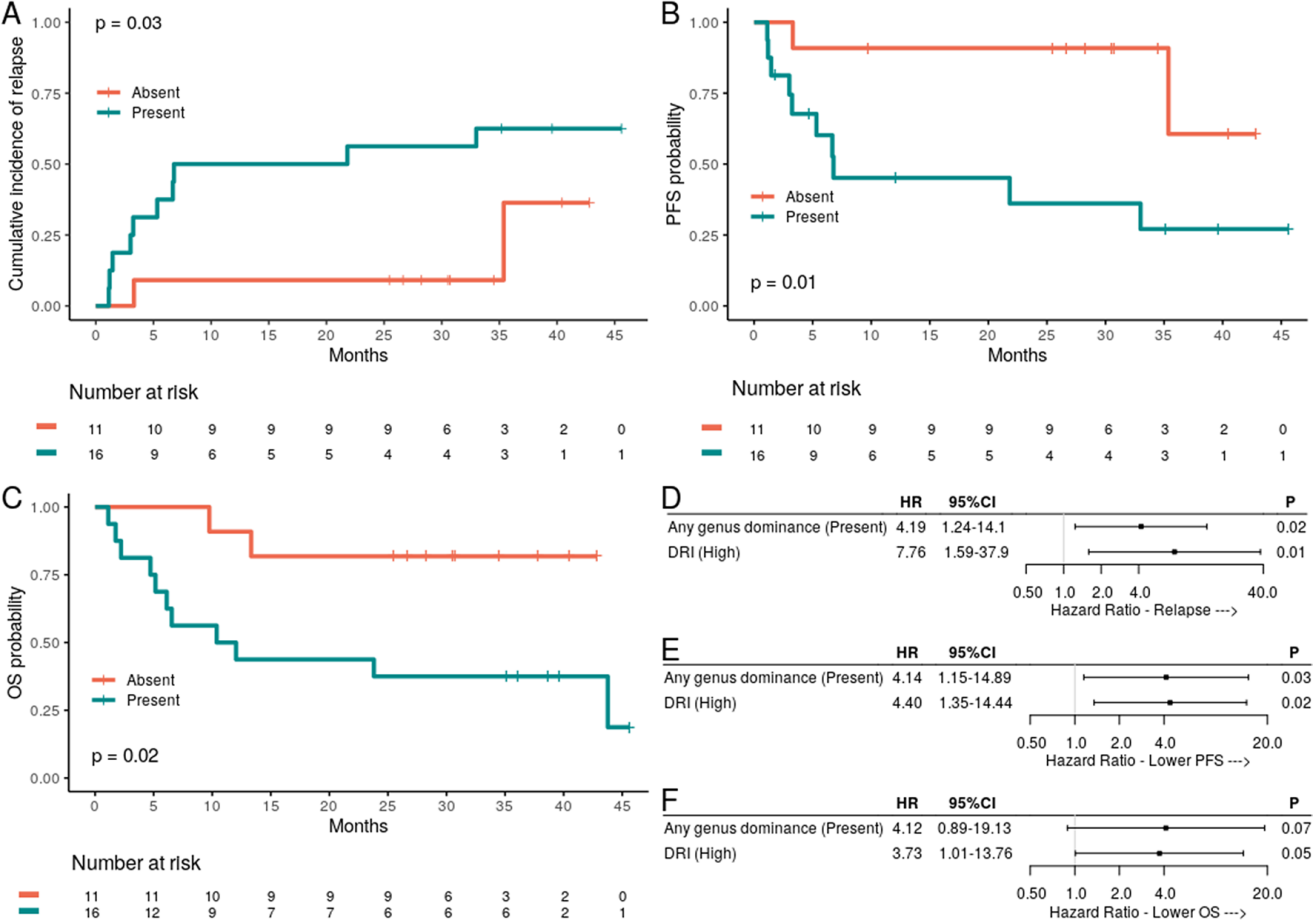
Association of any genus dominance with relapse, progression-free survival, and overall survival. (**A**) Cumulative incidence of relapse with patients (n = 27) stratified by any genus dominance at preconditioning. (**B**) Progression-free survival (PFS) with patients (n = 27) stratified by any genus dominance at preconditioning. (**C**) Overall survival (OS) with patients (n = 27) stratified by any genus dominance at preconditioning. (**D**) The DRI-adjusted hazard ratio for the association of dominance (relative abundance > 30%) of any genus at preconditioning and relapse (n = 27). (**E**) The DRI-adjusted hazard ratio for the association of dominance (relative abundance > 30%) of any genus at preconditioning and PFS (n = 27). (**F**) The DRI-adjusted hazard ratio for the association of dominance (relative abundance > 30%) of any genus at preconditioning and OS (n = 27).

Notably, 16 (59%) patients presented some type of bacterial dominance at preconditioning. Such events encompassed 4 different genera, all of which are oral commensal: *Streptococcus* (dominant in 9/16 patients) and *Veillonella* (dominant in 2/16 patients), both members of the Firmicutes phylum; *Neisseria* (dominant in 3/16 patients) and *Rothia* (dominant in 2/16 patients). Genus dominance was detected even among patients classified as having high diversity at preconditioning (Fig. 2A). The presence of dominance by any genus at preconditioning was also associated with an increased risk of relapse at 3 years when compared with the absence of dominance (63% versus 36%, respectively; HR 4.59, 95% CI 1.11–19, *P* = 0.03; Fig. 3A). When evaluating dominance by specific genera or types of genera at preconditioning, neither dominance by *Streptococcus* (56% versus 39%, respectively; HR 1.64, 95% CI 0.52–5.14, *P* = 0.4), nor dominance by facultative anaerobic genera (*Streptococcus* or *Rothia*; 56% versus 39%, respectively; HR 2.05, 95% CI 0.67–6.27, *P* = 0.21) were associated with an increased risk of relapse. Due to the very unequal group sizes, we could not evaluate the association between dominance by *Rothia* (2/27 patients), *Veillonella* (the only dominant anaerobe; 2/27 patients) or *Neisseria* (the only dominant aerobe; 3/27 patients) at preconditioning and the risk of relapse.

Additionally, the presence of dominance by any genus at preconditioning was associated with inferior PFS (19% versus 55%, respectively; HR 4.75, 95% CI 1.78–12.7, *P* = 0.01; Fig. 3B) and OS (38% versus 81%, respectively; HR 4.73, 95% CI 1.59–14.08, *P* = 0.02; Fig. 3C). No differences in aGVHD at 100 days (43% versus 63%, respectively; HR 0.50, 95% CI 0.18–1.37, *P* = 0.18), cGVHD at 3 years (19% versus 18%, respectively; HR 1.07, 95% CI 0.19–5.93, *P* = 0.94), or NRM at 3 years (20% versus 9%, respectively; HR 2.35, 95% CI 0.27–20.60, *P* = 0.44) were observed.

As expected, we also observed that patients with a high DRI had a significantly higher risk of relapse/progression, as compared with those with low-intermediate DRI at 3 years (62% versus 12%, respectively; HR 10.2, 95% CI 2.24–46.7, *P* < 0.01) and worse OS (77% versus 30%, respectively; HR 4.07, 95% CI 1.38–11.97, *P* = 0.01).

After adjusting analyses for the DRI, there was a trend toward a higher risk of relapse/progression in those with low OM diversity (HR 0.30, 95% CI 0.08–1.09, *P* = 0.07; Fig. 2C), and bacterial dominance of any genus remained significantly associated with the risk of relapse (HR 4.19, 95% CI 1.25–14.1, *P* = 0.02; Fig. 3D) and worse PFS (HR 4.14, 95% CI 1.15–14.89, *P* = 0.03; Fig. 3E). There was also a trend for bacterial dominance of any genus to be associated with worse OS (HR 4.12, 95% CI 0.89–19.13, *P* = 0.07; Fig. 3F).

Other relevant clinical parameters, such as conditioning intensity, underlying disease, and graft source, were not significantly associated with the risk of relapse (Fig. S5, Table S5).

### Genus presence and transplant outcomes

**Figure 4:**
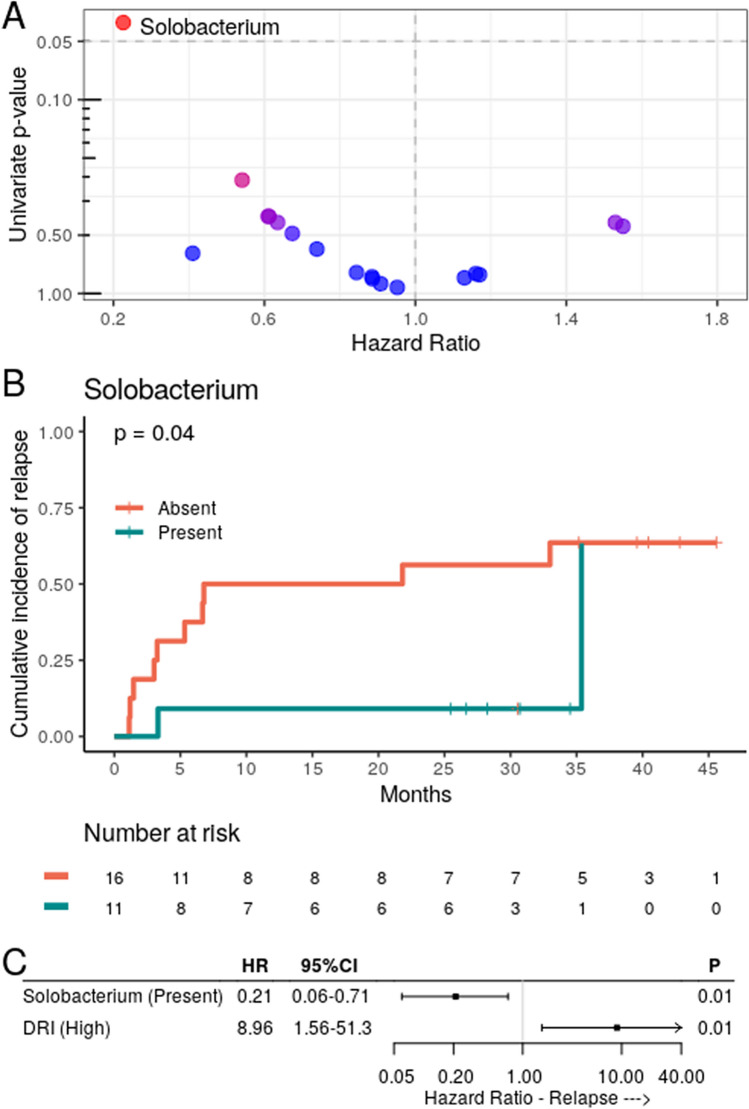
*Solobacterium* absence at preconditioning was associated with an increased risk of relapse in patients who underwent allo-HSCT. (**A**) Volcano plot for the univariate competing risk analysis of the association of relapse with the presence of specific genera at preconditioning (*P* value versus hazard ratio). The *Solobacterium* data point is indicated as it was the only genus significantly associated with relapse (*P* < 0.05). (**B**) Cumulative incidence of relapse with patients (n = 27) stratified by *Solobacterium* presence. (**C**) The DRI-adjusted hazard ratio for the association of *Solobacterium* presence at preconditioning and relapse (n = 27).

As the genus level represents the most specific taxonomic level that still provides reliable taxonomic classification for V3–V4 amplicons, to further evaluate the association between preconditioning OM and transplant outcomes, we analyzed whether any non-core genus (those present in 25–75% of samples) was associated with a higher risk of relapse. In this exploratory analysis (without adjustment for multiple comparisons), of the 18 genera that matched the selection criteria tested in a univariate analysis for relapse (Fig. 4A, Fig. S6), only *Solobacterium* was significantly associated with lower relapse risk (9% versus 56%, respectively; HR 0.23, 95% CI 0.05–0.94, *P* = 0.04; Fig. 4B), and this association remained significant after adjusting for DRI (HR 0.20, 95% CI 0.06–0.67, *P* = 0.01; Fig. 4C). However, after adjusting for multiple comparisons using the Bonferroni correction, because of the limited statistical power of this study, the univariate association between *Solobacterium* presence and lower relapse risk lost significance (*P* = 0.72). The relative abundance of *Solobacterium* at preconditioning per patient is depicted in Fig. S7. No differences in the presence of *Solobacterium* were found in other outcomes (aGVHD at 100 days: 64% versus 44%, respectively [HR 1.84, 95% CI 0.68–4.95, *P* = 0.23]; cGVHD: 27% versus 13%, respectively [HR 2.41, 95% CI 0.43–13.4, *P* = 0.31]; PFS: 55% versus 37%, respectively [HR 0.83, 95% CI 0.31–0.83, *P* = 0.71]; and OS at 3 years: 55% versus 28%, respectively [HR 0.99, 95% CI 0.32–3.08, *P* = 0.99]).

### Antibiotic use

From one week before until the first day of the conditioning regimen, 4 (13%) patients received antibiotics. From the first day of the conditioning regimen until engraftment, 28 (93%) patients received antibiotics: 20 (67%) used cefepime, 16 (53%) meropenem, 14 (47%) vancomycin, and four (13%) piperacillin-tazobactam. The use of these antibiotics were not associated with the risk of relapse (Fig. S5). We could not analyze the association between the use of antibiotics before transplant (30 days before starting the conditioning regimen) and OM bacterial diversity because of the small number of patients who used antibiotics at that time point.

## Discussion

In this single-center observational study, we prospectively collected samples from the oral mucosa of patients who underwent allo-HSCT. To the best of our knowledge, this is the first study to evaluate the possible impact of the OM using ASVs on allo-HSCT outcomes. ASVs, which are read sequences denoised to single-nucleotide resolution, is a more reproducible and comprehensive technique with higher sensitivity and specificity than operational taxonomic units (OTU) in analyzing microbiota^44–46^. The OTU can identify bacteria at the genus level, while ASVs allow to distinguish bacteria at the species level, which could explain discrepancies between our findings when compared to previous studies.

We observed that patients who presented low OM diversity or dominance of any genus before conditioning had a significantly increased risk of relapse. The dominance of any genus was also associated with worse PFS and OS. Although a low oral microbiota diversity and the dominance of any genus are proxies for microbiota dysbiosis, the former was not associated with worse PFS and OS. Only 7 (25%) patients share the binomial: low OM diversity and dominance of any genus, or high OM diversity and absence of dominance. The dominance of a single genus may denote a deeper immune imbalance and could represent a more sensitive predictor of alloHSCT outcomes when compared with OM diversity.

The OM has different niches in the same environment and is as diverse as the IM. Previous studies evaluating OM and allo-HSCT have shown conflicting results, likely because of the use of low-resolution techniques for microbiota analysis and the small sample sizes. In one case series, there were no changes in OM after allo-HSCT. The most common oral organisms, e.g., *Streptococcus*, *Gemella*, and *Veillonella*, remained relatively stable after transplant^34^. However, another study showed a reduction in alpha diversity after allo-HSCT when compared with the pretransplant OM^35^, and this reduction was more pronounced in patients who developed oral mucositis^47^. Besides, we did not find any direct correlation between the use of antibiotics after conditioning and transplant outcomes, as other studies have shown for IM diversity^23,24^.

Recently, IM has attracted attention as a potential predictive marker for allo-HSCT outcomes. Previous studies have shown that low IM diversity is associated with a higher risk of mortality, but not with the risk of relapse^25–27^, diverging from our findings.

Higher risk of aGVHD in patients with low IM diversity^48^ and a higher risk of transplant-related mortality attributable to GVHD^26^ were also reported. In the oral mucosa samples analyzed in the current study, low OM diversity was associated with an increased risk of relapse but did not change the risk of mortality, aGVHD or cGVHD.

The dominance of a specific bacterial group in IM, *Eubacterium limosum*, has also been shown to be related to relapse and disease progression. In our series, the dominance of any genus was associated with a higher risk of relapse.

As opposed to what has been observed for IM^27^, all dominant genera at preconditioning reported herein are commensal organisms. Thus, it is unlikely that they all have detrimental roles in the allo-HSCT setting, being more plausible that the presence of dominance by any genus is a proxy for low diversity/dysbiotic OM.

Furthermore, the presence of *Solobacterium* in the OM before conditioning seems to have a protective effect against relapse. *S. moorei*, the only species in the *Solobacterium* genus, is normally associated with halitosis^49,50^ and endodontic infection^51,52^. However, in the allo-HSCT scenario, the lack of *Solobacterium* could be a marker of dysbiosis, pretransplant disease status, or previous treatments. Alternatively, this genus may also play a role as an immune mediator by producing hydrogen sulfide^49^, a metabolite associated with decreased oxidative stress and increased sensitivity to antibiotics^53^. Although, the low overall *Solobacterium* relative abundance even in patients where it was present makes the latter alternative more unlikely, this finding need to be validated in future studies.

A previous study analyzed the tongue microbiota in patients who underwent alloHSCT and compared it with community-dwelling adults. AlloHSCT patients have a lower tongue microbiota alpha diversity when compared to community adults. Moreover, the presence of *Staphylococcus haemolyticus* or *Ralstonia pickettii* was associated with a higher risk of mortality. Nevertheless, no relationship was observed between alpha diversity of the tongue microbiota and incidence of transplant complications^46^. A study of salivary microbiota showed a reduction in alpha diversity during the course of transplantation. Again, no correlation between salivary microbiota diversity and alloHSCT outcomes was found^54^. The discrepancies between these studies and our findings may be related to different sites of sample collections, and different distinct microbiome analysis techniques.

Our study had several limitations of a relatively small and heterogenous single-center transplant cohort. However, as observed in studies of IM, in our series, OM showed a significant correlation with relapse and may also provide valuable information on host-related microbial dysbiosis, providing a simple, reproducible technique for collection and analysis prior to transplantation.

In conclusion, in the current study, we focused on preconditioning samples in order to identify potential clinical effects of OM on allo-HSCT outcomes and observer that lower OM diversity was associated with a higher risk of relapse after allo-HSCT and dominance by a single genus was associated with a higher risk of relapse and worse survival after allo-HSCT.

Prospective trials and validation cohorts are needed to confirm these findings and to test whether early interventions to correct OM dysbiosis or more aggressive strategies to prevent relapse in OM dysbiotic patients, such as early immunosuppression withdrawal, maintenance therapy, or prophylactic donor lymphocyte infusions, could improve the predicted adverse outcome.

## Supplementary Information

Supplementary Information.

## Abbreviations

95% Cis: Ninety-five percent confidence intervals
aGVHD: Acute GVHD
allo-HSCT: Allogeneic stem cell transplantation
ASVs: Amplicon sequencing variants
cGVHD: Chronic GVHD
DRI: Disease risk index
GVHD: Graft versus host disease
IM: Intestinal microbiota
NRM: Non-relapse mortality
OM: Oral mucosa microbiota
OS: Overall survival
PFS: Progression-free survival
