# Supplementary material for: Disruption of the oral microbiota is associated with a higher risk of relapse after allogeneic hematopoietic stem cell transplantation
Source: Sci Rep. 2021 Sep 2;11:17552. doi: 10.1038/s41598-021-96939-8 (PMC8413296; doi:10.1038/s41598-021-96939-8)
Supplement: Supplementary file 1 — Supplementary Information. [file 41598_2021_96939_MOESM1_ESM.docx]

# SUPPLEMENTARY MATERIAL

## Tables

**Table S1: Underlying disease, disease status at preconditioning, and oral microbiota diversity at preconditioning of 31 patients who underwent an allo-HSCT.**

|  | **OM diversity  at preconditioning** | **Diagnosis** | **Diasease status  at preconditioning** |
| --- | --- | --- | --- |
| 1 | Low | ALL | Refractory |
| 2 | High | ALL | CR2 |
| 3 | NA | ALL | CR1 |
| 4 | Low | ALL | CR2 |
| 5 | Low | ALL | CR3 |
| 6 | Low | ALL | CR1 |
| 7 | High | ALL | CR1 |
| 8 | NA | AML | CR1 |
| 9 | Low | AML | CR1 |
| 10 | High | AML | CR1 |
| 11 | Low | AML | CR1 |
| 12 | Low | AML | CR1 |
| 13 | High | AML | CR1 |
| 14 | Low | AML | CR1 |
| 15 | NA | AML | CR1 |
| 16 | High | AML | CR1 |
| 17 | High | AML | CR1 |
| 18 | Low | AML | Refractory |
| 19 | High | CLL | PR |
| 20 | Low | CML | CR2 |
| 21 | Low | MDS | CR1 |
| 22 | High | MDS | CR1 |
| 23 | High | MDS | CR1 |
| 24 | High | MDS | CR1 |
| 25 | High | MM | CR2 |
| 26 | High | NHL | Refractory |
| 27 | High | NHL | CR3 |
| 28 | Low | NHL | Refractory |
| 29 | Low | NHL | CR2 |
| 30 | Low | NHL | PR |

OM, oral microbiota; ALL, acute lymphoblastic leukemia; AML, acute myeloid leukemia; CLL, chronic lymphocytic leukemia; CML, chronic myeloid leukemia; MDS, myelodysplastic syndrome; MM, multiple myeloma; NHL, non-hodgkin lymphoma; CR1, first complete remission; CR2, second complete remission; CR3, third complete remission; PR, partial remission; NA, not available.

**Table S2: Correlation of relative abundance changes from preconditioning to engraftment and allo-HSCT outcomes.**

|  | **N** | **Present** | **Absent** | **P value** |
| --- | --- | --- | --- | --- |
| **aGVHD** |  |  |  |  |
| Enterococcus | 25 | 14 | 11 | 0.54 |
| Lactobacillus | 25 | 17 | 8 | 0.14 |
| Mycoplasma | 25 | 8 | 17 | 0.96 |
| Staphylococcus | 25 | 10 | 15 | 0.26 |
| **saGVHD** |  |  |  |  |
| Enterococcus | 25 | 14 | 11 | 0.52 |
| Lactobacillus | 25 | 17 | 8 | 1.00 |
| Mycoplasma | 25 | 8 | 17 | 0.42 |
| Staphylococcus | 25 | 10 | 15 | 0.58 |
| **cGVHD** |  |  |  |  |
| Enterococcus | 25 | 14 | 11 | 0.03 |
| Lactobacillus | 25 | 17 | 8 | 0.63 |
| Mycoplasma | 25 | 8 | 17 | 0.46 |
| Staphylococcus | 25 | 10 | 15 | 0.36 |
| **NRM** |  |  |  |  |
| Enterococcus | 25 | 14 | 11 | 0.19 |
| Lactobacillus | 25 | 17 | 8 | 0.10 |
| Mycoplasma | 25 | 8 | 17 | 0.12 |
| Staphylococcus | 25 | 10 | 15 | 0.67 |
| **Relapse** |  |  |  |  |
| Enterococcus | 25 | 14 | 11 | 0.78 |
| Lactobacillus | 25 | 17 | 8 | 0.74 |
| Mycoplasma | 25 | 8 | 17 | 0.19 |
| Staphylococcus | 25 | 10 | 15 | 0.09 |
| **PFS** |  |  |  |  |
| Enterococcus | 25 | 14 | 11 | 0.53 |
| Lactobacillus | 25 | 17 | 8 | 0.61 |
| Mycoplasma | 25 | 8 | 17 | 0.36 |
| Staphylococcus | 25 | 10 | 15 | 0.06 |
| **OS** |  |  |  |  |
| Enterococcus | 25 | 14 | 11 | 0.31 |
| Lactobacillus | 25 | 17 | 8 | 0.80 |
| Mycoplasma | 25 | 8 | 17 | 0.43 |
| Staphylococcus | 25 | 10 | 15 | 0.34 |

Only patients with preconditioning and engraftment samples were included (n = 25). The relative abundance of a genus was considered to increase during allo-HSCT for a given patient when the relative abundance at engraftment was greater than at preconditioning and the final relative abundance was ≥0.1%. GVHD, graft versus host disease; aGVHD, acute GVHD; saGVHD, severe acute GVHD; cGVHD, chronic GVHD; NRM, non-relapse mortality; PFS, progression-free survival; OS, overall survival.

**Table S3: Correlations of bacterial diversity with clinical features and transplant outcomes.**

|  | **Preconditioning (n = 27)** | | | **Aplasia (n = 28)** | | | **Engraftment (n = 26)** | | |
| --- | --- | --- | --- | --- | --- | --- | --- | --- | --- |
|  | **Low** | **High** | **P value** | **Low** | **High** | **P value** | **Low** | **High** | **P value** |
| **Sex** |  |  |  |  |  |  |  |  |  |
| Female | 9 | 4 |  | 8 | 6 |  | 4 | 8 |  |
| Male | 5 | 9 | 0.13 | 6 | 8 | 0.71 | 9 | 5 | 0.24 |
| **Underlying disease** |  |  |  |  |  |  |  |  |  |
| Acute leukemia | 9 | 6 |  | 7 | 9 |  | 9 | 6 |  |
| Other | 5 | 7 | 1 | 7 | 5 | 0.70 | 4 | 7 | 0.43 |
| **HCT-CI** |  |  |  |  |  |  |  |  |  |
| 0 | 7 | 9 |  | 8 | 8 |  | 4 | 9 |  |
| 1–2 | 5 | 1 |  | 3 | 3 |  | 5 | 3 |  |
| ≥3 | 2 | 3 | 0.24 | 3 | 3 | 1 | 4 | 1 | 0.15 |
| **Disease risk index** |  |  |  |  |  |  |  |  |  |
| Low–intermediate | 6 | 8 |  | 8 | 7 |  | 9 | 6 |  |
| High | 8 | 5 | 0.45 | 6 | 7 | 1 | 4 | 7 | 0.43 |
| **Conditioning intensity** |  |  |  |  |  |  |  |  |  |
| Reduced intensity | 8 | 8 |  | 10 | 7 |  | 7 | 9 |  |
| Myeloablative | 6 | 5 | 1 | 4 | 7 | 0.44 | 6 | 4 | 0.69 |
| **Total body irradiation** |  |  |  |  |  |  |  |  |  |
| No | 8 | 10 |  | 8 | 10 |  | 7 | 9 |  |
| Yes | 6 | 3 | 0.42 | 6 | 4 | 0.69 | 6 | 4 | 0.69 |
| **T-cell depletion** |  |  |  |  |  |  |  |  |  |
| No | 6 | 6 |  | 7 | 6 |  | 8 | 6 |  |
| Yes | 8 | 7 | 1 | 7 | 8 | 1 | 5 | 7 | 0.70 |
| **Graft source** |  |  |  |  |  |  |  |  |  |
| Peripheral blood | 10 | 9 |  | 10 | 10 |  | 8 | 8 |  |
| Bone marrow | 4 | 4 | 1 | 4 | 4 | 1 | 5 | 5 | 1 |
| **Donor** |  |  |  |  |  |  |  |  |  |
| Haploidentical | 5 | 3 |  | 5 | 4 |  | 4 | 5 |  |
| Matched sibling | 3 | 5 |  | 4 | 4 |  | 3 | 5 |  |
| Matched unrelated | 5 | 4 |  | 4 | 5 |  | 4 | 3 |  |
| Mismatched unrelated | 1 | 1 | 0.84 | 1 | 1 | 1 | 1 | 1 | 0.88 |
| **GVHD prophylaxis** |  |  |  |  |  |  |  |  |  |
| CsA + MTX | 3 | 6 |  | 4 | 5 |  | 6 | 2 |  |
| CsA + MMF | 7 | 4 |  | 6 | 5 |  | 2 | 8 |  |
| CsA + MMF + CyPT | 4 | 3 | 0.49 | 4 | 4 | 1 | 5 | 3 | 0.09 |
| **aGVHD** |  |  |  |  |  |  |  |  |  |
| No | 8 | 5 |  | 7 | 7 |  | 7 | 6 |  |
| Yes | 6 | 8 | 0.45 | 7 | 7 | 1 | 6 | 7 | 1 |
| **saGVHD** |  |  |  |  |  |  |  |  |  |
| No | 9 | 12 |  | 11 | 11 |  | 10 | 11 |  |
| Yes | 5 | 1 | 0.16 | 3 | 3 | 1 | 3 | 2 | 1 |
| **cGVHD** |  |  |  |  |  |  |  |  |  |
| No | 13 | 9 |  | 12 | 11 |  | 10 | 10 |  |
| Yes | 1 | 4 | 0.16 | 2 | 3 | 1 | 3 | 3 | 1 |
| **Oral mucositis** |  |  |  |  |  |  |  |  |  |
| No | 8 | 7 |  | 8 | 8 |  | 8 | 7 |  |
| Yes | 6 | 6 | 1 | 6 | 6 | 1 | 5 | 6 | 1 |
| **Febrile neutropenia** |  |  |  |  |  |  |  |  |  |
| No | 0 | 2 |  | 2 | 0 |  | 0 | 2 |  |
| Yes | 14 | 11 | 0.22 | 12 | 14 | 0.48 | 13 | 11 | 0.48 |
| **Age (years)** |  |  | 0.30 |  |  | 0.87 |  |  | 0.68 |

Diversity classification was based on the median Shannon index diversity measured across the study population at each collection time. Association of diversity classification and clinical parameters was evaluated by Fisher's exact tests for categorical variables and two-sided Student's t-tests for age. HCT-CI, hematopoietic cell transplantation-specific comorbidity index; CsA, cyclosporin A; MMF, mycophenolate mofetil; MTX, methotrexate; aGVHD, acute GVHD; saGVHD, severe acute GVHD; cGVHD, chronic GVHD.

**Table S4: Univariate competing risk analysis for the association of relapse with oral mucosa diversity.**

|  | **HR (95% CI)** | ***P* value** |
| --- | --- | --- |
| **Diversity at preconditioning (High)** | 0.27 (0.07–0.97) | 0.04 |
| **Diversity at aplasia (High)** | 1.30 (0.43–3.90) | 0.64 |
| **Diversity at engraftment (High)** | 0.73 (0.21–2.53) | 0.62 |

HR, hazard ratio; CI, confidence interval.

**Table S5: Univariate competing risk analysis for the association of relapse with clinical parameters.**

|  | **HR (95% CI)** | ***P* value** |
| --- | --- | --- |
| **Age (years)** | 0.97 (0.94–1.01) | 0.14 |
| **Underlying disease (AL versus other)** | 0.80 (0.25–2.56) | 0.70 |
| **HCT-CI (1–2 versus 0)** | 1.97 (0.63–6.21) | 0.25 |
| **HCT-CI (≥ 3 versus 0)** | 0.35 (0.05–2.76) | 0.32 |
| **DRI (High)** | 10.2 (2.24–46.7) | < 0.01 |
| **Conditioning intensity (Myeloablative)** | 0.95 (0.32–2.84) | 0.93 |
| **TBI (Yes)** | 2.04 (0.68–6.16) | 0.21 |
| **T-cell depletion (Yes)** | 2.43 (0.79–7.53) | 0.12 |
| **Graft source (Bone marrow)** | 0.96 (0.31–2.95) | 0.94 |
| **Donor (MSD versus haploidentical)** | 0.71 (0.13–3.91) | 0.69 |
| **Donor (MUD versus haploidentical)** | 1.97 (0.52–7.47) | 0.32 |
| **Donor (MMUD versus haploidentical)** | 15.6 (2.21–110) | < 0.01 |
| **GVHD prophylaxis (MMF versus MTX)** | 0.81 (0.23–2.90) | 0.75 |
| **GVHD prophylaxis (MMF+CyPT versus MTX)** | 0.69 (0.18–2.73) | 0.60 |
| **Tazobactam (Yes)** | 0.48 (0.07–3.28) | 0.46 |
| **Cefepime (Yes)** | 0.51 (0.17–1.53) | 0.23 |
| **Meropenem (Yes)** | 1.11 (0.35–3.49) | 0.86 |
| **Oral mucositis** | 1.41 (0.47–4.17) | 0.54 |

All GVHD prophylaxis protocols included CsA. HCT-CI, hematopoietic cell transplantation-specific comorbidity index; MMF, mycophenolate mofetil; MTX, methotrexate; TBI, total body irradiation; AL, acute leukemia; DRI, Disease Risk Index; MSD, matched sibling donor; MUD, matched unrelated donor; MMUD, mismatched unrelated donor; HR, hazard ratio; CI, confidence interval.

## Figures legends

**Figure S1: Bacterial richness within the oral mucosa decreases during allo-HSCT.** (A) Oral mucosa bacterial richness boxplot at preconditioning (n = 27), aplasia (n = 28), and engraftment (n = 26), as measured by the number of observed ASVs. Mann-Whitney U tests were used with the preconditioning collection as the reference for comparisons. The boxes highlight the median values and cover the 25th and 75th percentiles, with whiskers extending to the more extreme value within 1.5 times the length of the box. Outliers are represented explicitly. Asterisks represent statistical significance: *, *P* < 0.05; **, *P* < 0.01. ASV, amplicon sequencing variant.

**Figure S2: Changes in bacterial taxa during allo-HSCT.** Relative abundances of phyla (A), classes (B), orders (C), families (D) and genera (E) in the oral mucosa across transplantation phases for all patients (n = 30). Only taxa showing relative abundance ≥30% in at least one study sample or relative abundance ≥5% in at least 25% of study samples are shown. P, preconditioning; A, aplasia; E, engraftment.

**Figure S3: Significant changes in bacterial genera during allo-HSCT.** Significant genera relative abundance variations from preconditioning to aplasia and from preconditioning to engraftment according to ANCOM test (W > 0.7). Relative differences are represented by the log_2_-transformed average relative abundance fold change between groups.

**Figure S4: Relative abundance changes from preconditioning to engraftment of potentially pathogenic genera.** Each line represents a study patient. Only patients with preconditioning and engraftment samples were included (n = 25). The relative abundance of a genus was considered to increase during allo-HSCT for a given patient when the relative abundance at engraftment was greater than at preconditioning and the final relative abundance was ≥0.1%.

**Figure S5: Univariate competing risk analysis for the association of relapse with clinical parameters**. The variables are sorted in ascending order according to the hazard ratio. All GVHD prophylaxis protocols included CsA. HCT-CI, hematopoietic cell transplantation-specific comorbidity index; MMF, mycophenolate mofetil; MTX, methotrexate; TBI, total body irradiation; AL, acute leukemia; DRI, Disease Risk Index; CI, conditioning intensity; MSD, matched sibling donor; MUD, matched unrelated donor; MMUD, mismatched unrelated donor.

**Figure S6: Univariate competing risk analysis for the association of relapse with specific genus presence at preconditioning**. The variables are sorted in ascending order according to hazard ratio. Only genera present in at least 25% of samples and absent in at least 25% of samples were evaluated.

**Figure S7: OM Solobacterium relative abundance in preconditioning samples.** Each bar represents a study patient.

## Figures

**Figure S1**


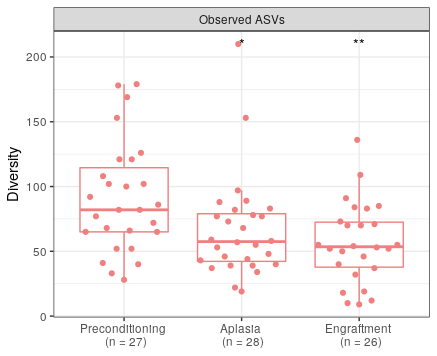


**Figure S2**


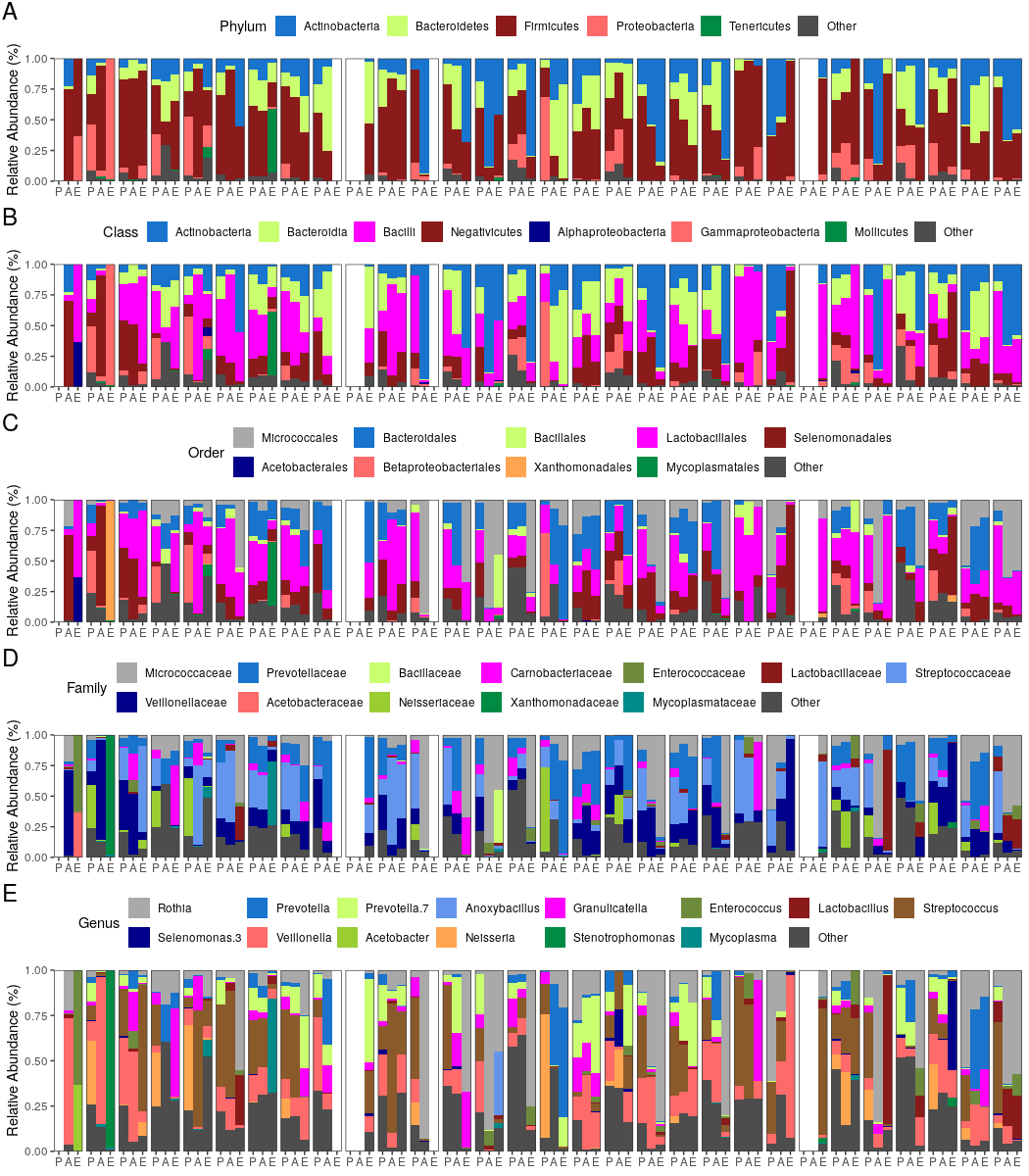


**Figure S3**

**
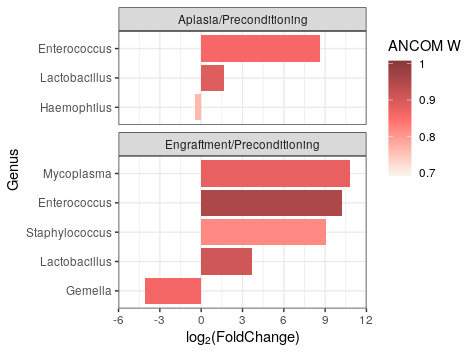
**

**Figure S4**

**
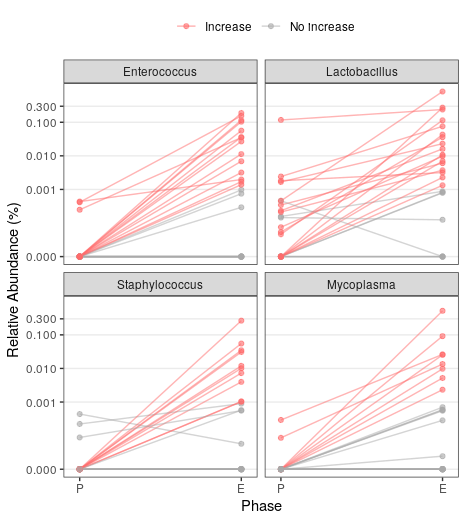
**

**Figure S5**

**
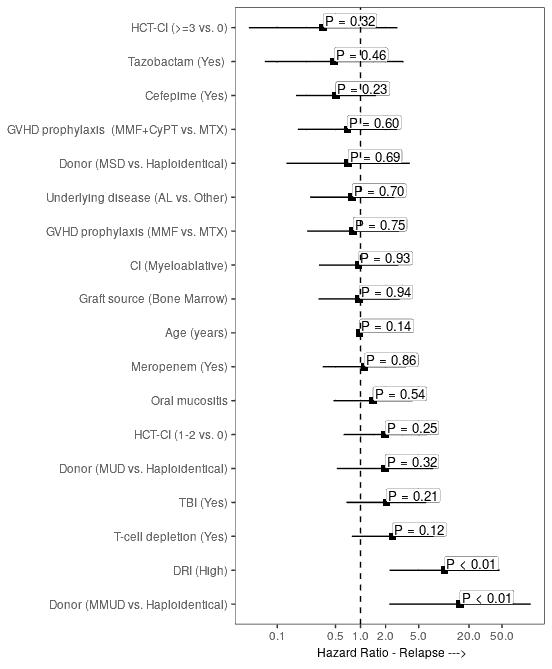
**

**Figure S6**

**
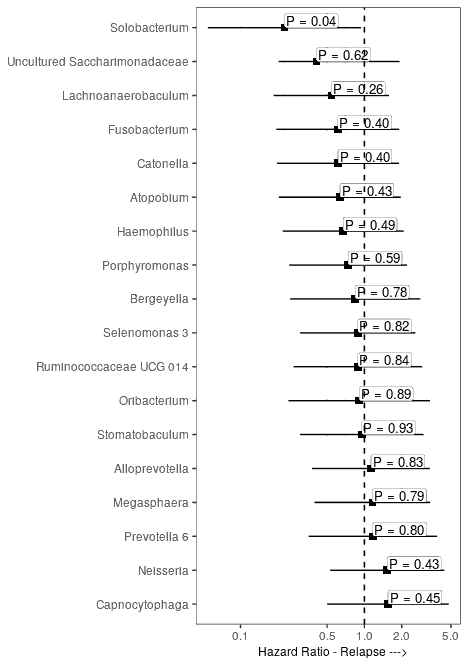
**

**Figure S7**

**
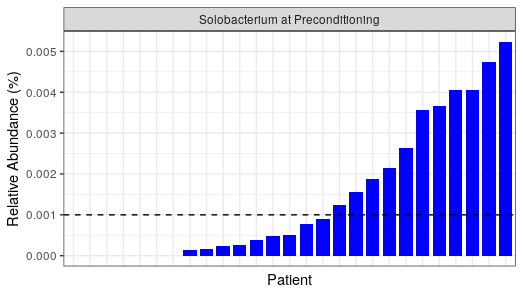
**
